# Primary Health Centre disaster preparedness after the earthquake in Padang Pariaman, West Sumatra, Indonesia

**DOI:** 10.1186/1756-0500-4-81

**Published:** 2011-03-25

**Authors:** Ahmad Fuady, Trevino A Pakasi, Muchtaruddin Mansyur

**Affiliations:** 1 Department of Community Medicine, Faculty of Medicine, Universitas Indonesia, Jakarta, Indonesia

## Abstract

**Background:**

The West Sumatra earthquake that occurred on September 30, 2009, caused severe damage in some districts, including Padang Pariaman. As Padang Pariaman is an earthquake-prone area, disaster and emergency management is necessary. Due to the limited health facilities, the health services completely rely on Puskesmas (Primary Health Centres, PHCs). This study is aimed at assessing the preparedness of PHCs to response to potential disasters in their surrounding area.

**Findings:**

Padang Pariaman district was used in a case study setting to assess the readiness and preparedness of the PHCs there to face disasters. Self-administered questionnaire, key informant interview, and direct observation were used to obtain the data on human resources, facilities preparedness, and the procedures. The investigation focused on measuring four aspects, i.e. human resources, facilities preparedness, standard operating procedure (SOP), and policy. Due to the limited co-operation of the head of the PHCs, three PHCs were directly observed as a subsample. The evaluation was performed six months after the impact phase of the earthquake and three months after the PHCs' health staff training on improving the primary health care services. The number and quality of health staff in Padang Pariaman was far below ideal. Fewer than half of the PHCs had emergency facilities and only one considered the need for triage and fire management, whereas the transportation mode was still limited. An SOP and policy for facing disasters were not available in any of the PHCs. Therefore, promoting disaster preparedness, technical provision, including health staff training, is necessary.

**Conclusions:**

Padang Pariaman district has not yet prepared its PHCs to face disaster, so it is apparent that PHCs' disaster preparedness in Padang Pariaman and also other earthquake-prone areas in Indonesia should be promoted. This should include increasing the number of doctors, providing training for health staff, and developing a comprehensive approach as well as coordination among government, hospitals, PHCs, and NGO's for disaster preparedness.

## Findings

### Background

A 7.6-magnitude earthquake occurred in West Sumatra in the evening of September 30, 2009. It caused severe damage in several districts, especially Padang and Padang Pariaman. Padang Pariaman district was one of the worst hit by the earthquake, with a large number of fatalities. Approximately 675 people died (1.74/1000 population) in Padang Pariaman district, followed by 313 people in Padang city (0.38/1000 population). In addition, about 86 primary health centres (PHCs) buildings were damaged that further exacerbated the problems related to patient care.

**Figure 1:**
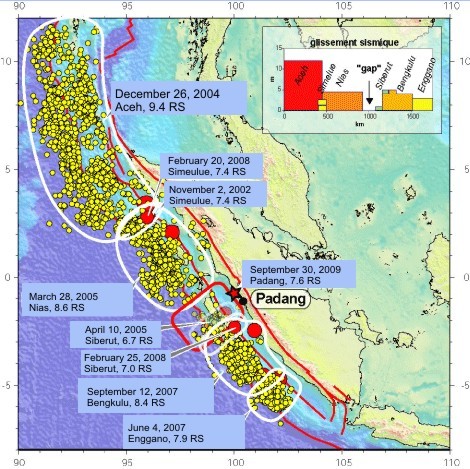
**Earthquake-prone area in west coast of Sumatra island**. Modified from Vigny [2]. Red dots show large subduction earthquake epicentre, yellow dots depict the aftershocks occurring within one month after the main shock. The red star shows the epicentre of September 30th, Padang earthquake. The inset box, shows the amount of average slip (in meters) associated to each earthquake since 2000 and the obvious slip deficit in front of Padang, which will have to catch up with the remaining of the subduction sooner or later, in successive medium-size events or in one larger.

As the west coast of Sumatra Island is prone to earthquakes, the latest one occurred on the Sumatran subduction[1]. An examination of that particular list and series of earthquake events among the islands of the west coast of Sumatra (see Figure 1)[2] highlights the urgent need for a disaster management program for the affected areas. In this situation, a disaster management program has to be considered to avoid or lessen the impact of disasters through mitigation, preparedness, response, and recovery[3-5].

There is only one state district hospital in Padang Pariaman, and it is located on the outskirt of the district in Padang Pariaman city (see Table 1). This single hospital has to serve 17 subdistricts, 46 Nagari (villages), and 364 Korong (sub-villages). Due to the limited health facilities, the health services rely completely on Puskesmas (Primary Health Centres, PHCs) that are a state-run, public health service that is responsible for providing health care in the sub-district area. The PHCs' capacity regarding disaster and emergency preparedness should be strengthened. This preparedness includes human resources, facilities preparedness, standard operating procedure (SOP), and policy for facing disasters, particularly earthquakes. This study is aimed at assessing the preparedness of PHCs to respond to potential disasters in their surrounding area.

**Table 1:** Health facilities in Padang Pariaman district

Health facilities	Coverage area	Number
District hospitals	District	1
Private hospitals	District	0
PHCs	Sub district	21
Supporting PHCs	Village	86

## Method

Using a case study, this study proposed to measure empirically the disaster preparedness of the PHCs in Padang Pariaman district. In order to ensure that data represented the objective figures for settled, recovered PHCs, the evaluation was performed in April 2010, approximately six months after the impact phase of the September 2009 earthquake and three months after the PHCs' health staff had received training on improving the primary health services.

There were nine surveyed PHCs, in Sungai Limau, Sungai Geringging, Batu Basa, Padang Alai, Patamuan, Sicincin, Pakandangan, Sintuk, and Ulakan. These nine out of 21 PHCs in Padang Pariaman district were chosen by the District Health Office to receive health staff training in capacity building in January 2010 by Faculty of Medicine, Universitas Indonesia. The nine PHCs were chosen as the sample due to their characteristics that were representative of PHC conditions in the district, the accessibility, the facility, as well as the coverage area. They cover ten out of 21 sub-districts (47%) and 150,878 of the 387,931 population (39%) in the district. The four days of lectures covered topics ranging from mother and child health, immunization programs, mental health, medical rehabilitation, and PHC management. The topics were prioritized on the basis of the result of a rapid needs assessment conducted in October 2009. The objective of this training was to improve PHCs' capacity for providing health care service for the community.

Six months after the earthquake, the PHCs' improvement was evaluated, and disaster preparedness was included in the evaluation of the PHCs' management. A self-administered questionnaire (see Additional file 1), key informant interview, and direct observation were used to obtain the data on human resources, facilities preparedness, and the procedures. The investigation focused on measuring four aspects, that were human resources, facilities preparedness, standard operating procedure (SOP), and policy.

Due to the limited co-operation of the head of the PHCs, three PHCs were directly observed as a subsample and the observations were attended by other PHCs on the next day to view the real condition in PHCs. The chosen PHCs were Sungai Limau (attended by Sungai Geringging, Padang Alai, and Batu Basa), Pakandangan (attended by Sintuk and Ulakan), and Sicincin (attended by Patamuan) that have better accessibility and facilities, including the remaining buildings after the earthquake, than other PHCs. Interviews were also conducted with several key informants in the PHCs during the observation to support the questionnaire data. Discussion about the strengths and the weaknesses of each PHC was also held.

The study was approved by the Research Ethical Committee of the Faculty of Medicine, Universitas Indonesia, Cipto Mangunkusumo Hospital, Jakarta, Indonesia, prior to being conducted.

## Results and Discussion

The nine PHCs had their own characteristics and were representative of the district. PHCs' characteristics based on coverage and available facilities can represent the data from other PHCs in Padang Pariaman. Based on the range of coverage, the nine selected PHCs represented PHC service from the low to high coverage (see Table 2). Nevertheless, the Ministry of Health for Republic of Indonesia have set the program standard of PHC to be applied throughout Indonesia. Table 2 shows that each of the PHCs had one district as their coverage area, except for the one in Sicincin due to its small population size. The percentage of damaged to Korongs was very high. Eight of the nine surveyed PHCs were also damaged, although the remaining one was still in a good condition. A similar situation applied to the water and electricity supply in the PHCs, and the earthquake damaged six of the nine PHCs' water facilities.

**Table 2:** Condition of PHCs after the earthquake

	Sungai Limau	Sungai Geringging	Batu Basa	Padang Alai	Patamuan	Sicincin	Pakandangan	Sintuk	Ulakan
*Coverage area*									
# of sub-districts	1	1	1	1	1	2	1	1	1
# of Nagaris (villages)	2	2	1	1	2	2	5	2	2
# of Korong (sub-villages)	18	5	6	9	14	8	27	29	33
# of damaged Korongs	3	5	6	9	8	8	27	25	28
%age of damaged Korongs	16.67%	100%	100%	100%	57.14%	100%	100%	86.21%	84.95%
# of population	29088	13974	19659	6187	15483	11619	18469	16456	19943
# of fatalities	23	2	0	84	132	9	6	1	12
*Facilities*									
Buildings	Damaged	Damaged	Damaged	Damaged	Damaged	Damaged	Damaged	Damaged	Good
Water supply	Damaged	Damaged	Damaged	Damaged	Damaged	Damaged	Good	Good	Good
Electricity	Damaged	Damaged	Damaged	Damaged	Damaged	Damaged	Good	Good	Good

The emergency preparedness in the PHCs relates to the processes involved in ensuring that the PHCs were well-prepared: to forecast disastrous events; to minimize the loss of life, injury, and damage to property; to provide rescue, relief and rehabilitation; as well as to improve the resources and capability to sustain the essential functions of the organization[6,7]. From the WHO pre-disaster preparedness activities guidance, we have developed a simple assessment to determine the PHCs' degree of disaster preparedness in four aspects, they were human resources, facilities preparedness, SOP or existing programs related to disaster, and policy.

Although the human resources were more closely related to the development of PHCs, these were vital when facing disaster. The human resources were assessed by the physician to population ratio and the given training. The physician to population ratio was based on the availability of physicians in an area. The ideal ratio need of physicians to the population in an area is 1:2500, according to the Ministry of Health of Indonesia in 2010[8]. Table 3 shows that Padang Pariaman district lacks health staff to support PHCs services, that can be further exacerbated when a disaster occurs. This should be some concern of the government. Not only can they provide a good service, but the ideal number of physicians in an area is a vital part of disaster preparedness, particularly in disaster-prone areas. This problem is also about the maldistribution of physicians between the urban and rural areas. To solve the problem, a new policy for the distribution of physicians, that is both clearer and can be more consistently applied, is needed[8,9].

**Table 3:** Human resources in the PHCs

	Sungai Limau	Sungai Geringging	Batu Basa	Padang Alai	Patamuan	Sicincin	Pakandangan	Sintuk	Ulakan
*Number*									
# of physicians	3	1	2	1	1	3	1	0	1
# of midwives	21	10	14	9	12	16	18	17	21
# of nurses	4	6	2	6	6	4	3	8	6
Physician:Population Ratio	1 : 9696	1 : 13974	1 : 9829	1 : 6187	1 : 15483	1 : 3873	1 : 18694	0	1 : 19943
Midwife:Population Ratio	1 : 1385	1 : 1397	1 : 1404	1 : 687	1 : 1290	1 : 726	1 : 1039	1 : 968	1 : 949
Nurse:Population Ratio	1 : 7272	1 : 2329	1 : 9829	1 : 1031	1 : 2580	1 : 2905	1 : 6231	1 : 2057	1 : 3323
*Training received by health staff*									
Disaster preparedness	Yes	No	No	No	Yes	No	No	No	No
Basic life support	No	No	No	No	Yes	No	No	No	No
Advance life support	No	No	No	No	No	No	No	No	No
Public health emergency	Yes	No	No	No	No	No	No	No	No

The health workers in the PHCs should also be provided with sufficient competency to face disasters. There are some competencies that health workers require in order to be ready to cope with disasters, such as disaster preparedness training, basic and advanced life support, as well as separate public health emergency training[10]. We have separated public health emergency training from disaster preparedness training due to its broader aspect including bioterrorism, the appearance of infectious agent or biological toxin, and chemical attack. Four basic training issues were assessed. Only two of the nine PHCs had sent their staff for disaster preparedness training. Only one PHC had trained its health staff in basic life support and none of the PHCs' health staff had received any advanced life support training, whereas only one PHC had attended a public health emergency training. Based on observation data, it was concluded that some of the heads of the PHCs were unaware of disaster preparedness training, basic and advanced life support training, as well as public health emergency training. This was a basic problem, because every physician who was responsible for managing the healthcare in a sub-district had to possess these capabilities, not only for the health staff themselves, but also for teaching the community to give first aid after a disaster[11]. This may have arisen because of the lack of a human resources development program by the District and Provincial Health Offices.

The second assessment was intended to measure the facilities preparedness, including emergency response facilities and transportation mode. Only four of the nine PHCs had any emergency facilities at all. Only one PHC had considered the need for triage and fire management, and four of them did not have any prepared secure area or a generator in place in case of power failure. In order to be able to implement their functions, including the disaster preparedness, the PHCs should also have an available mode of transportation, which is vital to transporting health workers and disaster victims following a disaster. The PHCs in Padang Pariaman lacked transportation facilities in the form of ambulances, mobile PHCs, or motorcycles to cover their areas (see table 3). It was concluded that PHCs in Padang Pariaman were not ready to become disaster management facilities.

The SOP and policy that should be prepared for facing disasters were not available in any of the PHCs. After the earthquake, the PHCs implemented a disaster response by themselves and there was no clear coordination between the PHCs, NGOs, and the local government. The PHCs had not even thought about how the system will be built if disaster struck. This indicated that the PHCs in Padang Pariaman were not ready to serve as health facilities in the face of disasters and that there had been no effort to implement disaster preparedness procedures. Having observed several health centres, the need was identified to build the system, and support in doing so is required from the government and health organization policy.

## Conclusion

In this earthquake-prone area with its limited healthcare facilities, PHCs should be optimalized as facilities that form part of disaster response system. Six months after the earthquake, the PHCs in Padang Pariaman district still had not prepared their systems for facing disasters. Thus, it is vital to promote PHCs' disaster preparedness in Padang Pariaman district and also other earthquake-prone areas in Indonesia. This should include increasing the number of physicians, providing training for health staff, and developing a comprehensive approach as well as coordination amongst the government, hospitals, PHCs, and NGOs.

## List of Abbreviations used

**PHC**: Primary Health Centre, **SOP**: Standard Operating Procedure, **NGO**: Non-Government Organization.

## Competing interests

The authors declare that they have no competing interests.

## Authors' contributions

AF and TAP were responsible for the study concept and design. AF and TAP were responsible for the implementation of the study and the acquisition of the data. AF analyzed the data on the advice of TAP and MM. All of the authors drafted and critically revised the manuscript for important intellectual content. AF and MM obtained funding and are the guarantors. AF, TAP, and MM have read and approved the final manuscript.

## Supplementary Material

Additional file 1 **Questionnaire**. Click here for file
