# Supplementary material for: Primary Health Centre disaster preparedness after the earthquake in Padang Pariaman, West Sumatra, Indonesia
Source: BMC Res Notes. 2011 Mar 25;4:81. doi: 10.1186/1756-0500-4-81 (PMC3072331; doi:10.1186/1756-0500-4-81)
Supplement: Additional file 1 — Questionnaire. [file 1756-0500-4-81-S1.RTF]

MANUAL
EVALUASI PROGRAM PUSKESMAS
KABUPATEN PADANG PARIAMAN


Evaluasi program Puskesmas ini dilakukan kepada Puskesmas yang telah mengikuti pelatihan “Capacity Building Rehabilitasi Pasca Bencana Sumatera Barat” yang dilaksanakan FKUI pada bulan Januari 2010. 

Data yang diambil berupa data deskriptif dan ditulis dalam kolom 'Deskripsi'. Bila ada data program, contoh rencana kerja, dan sebagainya dapat disertakan sebagai lampiran (sesuai petunjuk dalam pengisian form).


Manual
Primary Health Center (PHC) Program Evaluation
Padang Pariaman District

This Primary Health Center (PHC) program evaluation is implemented to PHCs that had attended in training “Capacity Building and Rehabilitation Post West Sumatra Earthquake” that held by Faculty of Medicine Universitas Indonesia in Januari 2010. 

Description of the data should be written on 'Description' column. If there are any program data, examples of plan of action, etc, it should be attached in this form (as in guidance).
Nama Puskesmas/Name of PHC	:
Alamat/Adress			: 
Kepala Puskesmas/Head of PHC	: 
Nomor HP/Phone number	: 

No	Evaluasi/Evaluation	Deskripsi/Description	
INPUT	
1.	Cakupan wilayah kerja/Coverage area	
	Cakupan wilayah yang dilayani/served coverage area
a.	Kecamatan/sub-district
b.	Jumlah nagari/number of villages
c.	Jumlah korong/number of subvillage
d.	Jumlah korong yang rusak berat/number of damaged villages
e.	Jumlah penduduk/number of population
f.	Jumlah korban/number of victims	
___________________________
______________ nagari
______________ korong
______________ korong
______________ jiwa
______________ jiwa	
2.	Kondisi Puskesmas/PHC condition                                                        Jenis Kerusakan/level of damage	
	Kondisi bangunan dan fasilitas fisik/physical condition
a.	Bangunan/buildings
-	Deskripsikan/please describe:
____________________________________________
____________________________________________
____________________________________________
____________________________________________
____________________________________________
-	Berikan gambar/foto menyeluruh dan bagian yang rusak
Please attached the picture of damaged building(s)
-	Berikan foto gedung secara utuh dari jauh (50 m) 
Please attached the picture of building from 50 m.
b.	Sarana air bersih/water supply
c.	Listrik/electricity supply	
berat/sedang/ringan
severe/moderate/mild


	
	Transportasi/transportation mode
a.	Ambulance/ambulance
b.	Puskesmas keliling/mobile PHC
c.	Sepeda motor/motorcycle	
Ada/tidak ada , Rusak/tidak rusak
Available/Unavailable,Good/Damaged	
3.	Fasilitas/Facilities		
	Fasilitas pelayanan medik/medical facilities:
a.	IGD/emergency
b.	Poli umum/general policlinic
c.	Poli gigi/dental clinic
d.	Rawat inap: keperawatan, kebidanan/in ward facilities
e.	Promosi kesehatan/health promotion
f.	Kamar bersalin/delivery room
g.	Ruang KIA: ANC, PNC, KB/mother and child health room
h.	Gizi/nutrition
i.	Apotek/drugs room
j.	Rekam medik/medical record
k.	Lain-lain/Others
____________________________________
____________________________________
Deskripsi tambahan/additional description:
___________________________________________________
___________________________________________________
___________________________________________________
___________________________________________________
___________________________________________________
___________________________________________________

Fasilitas menghadapi bencana/disaster preparedness facilities:
a.	Triage/triage
b.	Gawat darurat/emergency
c.	Penanggulangan kebakaran/fire management
Jelaskan/description: ____________________________
____________________________________
____________________________________
____________________________________
d.	Area aman untuk berkumpul/secure area
Jelaskan/description: ____________________________
____________________________________
____________________________________
____________________________________
e.	Generator set/generator set	
Ada/tidak ada
Available/Unavailable


	
4.	Sumber Daya Manusia/Human resources	
	SDM 
Human resources	Sebelum gempa
Before the earthquake	Sesudah gempa/after	Pernah mendapat pelatihan*	
			Meninggal/died	Sakit/ill	Tetap bekerja/fit	Bencana	BLS	ALS	MH	PHE	
	a.	Dokter/physician
b.	Bidan/midwive
c.	Perawat/nurse
d.	Perawat gigi/dental nurse
e.	Sanitarian/environmental workers
f.	Gizi/nutritionist
g.	Lab analis/lab analyst
h.	Administrasi/administration staff
i.	Satpam/security
j.	Lainnya/others
.......................
.......................					


* Bencana= Kesiapan bencana/emergency preparedness
* BLS = Basic life support
* ALS = Advance life support
* MH = Mental health
* PHE = Public health emergency	
5	Kebijakan/Policy
a.	Aturan dasar/guidance
b.	Perangkat evaluasi/evaluation form
(Lampirkan sebagai contoh: Perangkat yang disusun dalam raker; penetapan masalah, prioritas, identifikasi penyebab, cara penyelesaian, indikator keberhasilan.)
(Please attach the example(s): planning, problem identificaton, priority, problem solving method, target, indicator)	
Ada/Tidak
Available/Unavalaible

	
6	Finansial/Budgeting 
a.	Sumber/Source


b.	Jumlah/tahun/Annual budget
c.	Kecukupan/
Jelaskan/description : _______________________________________
________________________________________________
________________________________________________ 
d.	Asuransi kesehatan/insurance	
- __________________________
- __________________________
- __________________________

Cukup/tidak cukup
- __________________________
- __________________________
- __________________________	
7	Sistem informasi manajemen Puskesmas (SIMPUS)/PHC information management system	Ada/tidak ada
Available/Unavailable	

PROSES/Process	
1	Perencanaan program/Program planning
a.	Perencanaan program wajib/compusary program planning
-	Menggunakan Gantt Chart: usulan dan POA/Using Gantt Chart 
(Lampirkan sebagai contoh)/ (Attach the example)
b.	Perencanaan program pengembangan/Extended program planning
-	Identifikasi : survey mawas diri dan Delbecq technique/
Identification: survey and Delbecq technique
-	Menggunakan Gantt Chart: usulan dan POA/Using Gantt Chart
(Lampirkan sebagai contoh)/(Attach the example)	

Ya/Tidak
Yes/No


	
2	Pelaksanaan dan pengendalian/Operating and Controlling
a.	Pengorganisasian/Organizing
-	Menggunakan Gantt Chart/Using Gantt Chart
-	Kerjasama lintas sektor/Multi-sector cooperation
b.	Penyelenggaraan/Operating
-	Kendali mutu/Quality 
-	Kendali biaya/Cost
c.	Pemantauan/Controlling
-	Lokakarya mini bulanan/Monthly evaluation
-	Lokakarya mini tribulanan/Three-monthly evaluation
d.	Penilaian/Assessing 
Jelaskan cara penilaian/Describe assessment method : 
________________________________________________
________________________________________________
________________________________________________
________________________________________________
________________________________________________
________________________________________________
________________________________________________
________________________________________________
________________________________________________	

Ya/Tidak
Yes/No


	
3	Pengawasan dan pertanggungjawaban/Evaluating 
________________________________________________
________________________________________________
________________________________________________
________________________________________________
________________________________________________
________________________________________________
________________________________________________
________________________________________________
	


	
4	Koordinasi/Coordination
a.	Kecamatan/sub district
b.	Dinkes Kabupaten/District Health Office
c.	Pelayan kesehatan tingkat pertama lainnya/Other primary health care
d.	Fasilitas rujukan (RS/RSUD)/referral facilities
e.	Lintas sektor/multi-sector
f.	Masyarakat/community
Efektivitas dan masalah/effectiveness and problem: ________________________________________________
________________________________________________
________________________________________________
________________________________________________
________________________________________________
________________________________________________
      ________________________________________________	(Jenis koordinasi dan frekuensi)
(type of coordination and its frequency)


	

OUTPUT	
1	Program yang berjalan/Running compulsary program
Program Wajib/compulsary program
1. Promosi Kesehatan (Promkes)/health promotion
·	Penyuluhan Kesehatan Masyarakat/community health promotion

·	Sosialisasi Program Kesehatan/health program socialization

·	Perawatan Kesehatan Masyarakat (Perkesmas)/community health nursing

2. Pencegahan Penyakit Menular (P2M)/infectious disease control :
·	Surveilens Epidemiologi/surveillance

·	Pelacakan Kasus : TBC, Kusta, DBD, Malaria, Flu Burung, ISPA, Diare, IMS (Infeksi Menular Seksual), Rabies/ case detection: tuberculosis, lepra, haemmorhagic dengue fever, avian influenza, diarrhea, sexual transmitted disease, rabies.
3. Program Pengobatan/treatment program :
·	Rawat Jalan Poli Umum/general policlinic
·	Rawat Jalan Poli Gigi/dental clinic
·	Unit Rawat Inap : Keperawatan, Kebidanan/in-ward facilities
·	Unit Gawat Darurat (UGD)/emergency
·	Puskesmas Keliling (Puskel)/mobile clinic
4. Kesehatan Ibu dan Anak (KIA) /mother and child health
·	ANC (Antenatal Care) , PNC (Post Natal Care), KB (Keluarga Berencana/family planning),
·	Persalinan,  Rujukan Bumil Resti, Kemitraan Dukun/delivery and partnership
5. Upaya Peningkatan Gizi/nutritional improvement program

6. Kesehatan Lingkungan/environmental health :
	Deskripsi /description


__________________________
__________________________
__________________________
__________________________
__________________________
__________________________
__________________________
__________________________
__________________________
__________________________
__________________________
__________________________
__________________________
__________________________
__________________________
__________________________
__________________________
__________________________
__________________________
__________________________
__________________________
__________________________
__________________________
__________________________
__________________________
__________________________
__________________________
__________________________
__________________________
__________________________
__________________________
__________________________
__________________________
__________________________
	
2	Program tambahan Puskesmas/Extended program(s)
Jelaskan/description: ___________________________________________
___________________________________________________
___________________________________________________
___________________________________________________
___________________________________________________
___________________________________________________
___________________________________________________
___________________________________________________
___________________________________________________
___________________________________________________
___________________________________________________
___________________________________________________
___________________________________________________	


	
